# Prehospital Pandemic Respiratory Infection Emergency System Triage score can effectively predict the 30-day mortality of COVID-19 patients with pneumonia

**DOI:** 10.1080/07853890.2024.2407954

**Published:** 2024-09-25

**Authors:** Chen Li, Liang Wu, Zhao Yang, Junyuan Tan, Xiaodong Jia, Kaili Wang, Haibin Su

**Affiliations:** a Senior Department of Hepatology, The Fifth Medical Center of PLA General Hospital, Beijing, China; b Senior Department of Oncology, The Fifth Medical Center of PLA General Hospital, Beijing, China; c Medical Service Department, The Fifth Medical Center of PLA General Hospital, Beijing, China

**Keywords:** Pandemic Respiratory Infection Emergency System Triage, Coronavirus disease 2019, mortality, physiological parameters, prehospital

## Abstract

**Background:**

Coronavirus disease 2019 (COVID-19) patients with pneumonia should receive the guidance of initial risk stratification and early warning as soon as possible. Whether the prehospital Pandemic Respiratory Infection Emergency System Triage (PRIEST) score can accurately predict the short-term prognosis of them remains unknown. Accordingly, we aimed to assess the performance of prehospital PRIEST in predicting the 30-day mortality of patients.

**Methods:**

This retrospective study evaluated the accuracy of five physiological parameters scores commonly used in prehospital disposal for mortality prediction using receiver operating characteristic curves and decision curve analysis. Cox proportional hazard regression analysis was conducted to evaluate independent predictors associated with the 30-day mortality.

**Results:**

A total of 231 patients were included in this study, among which 23 cases (10.0%) died within 30 days after admission. Compared with survivor patients, non-survivor patients had greater numbers of comorbidities, signs and symptoms, complications, and physiological parameters scores and required greater prehospital care (*p* < 0.05). When the PRIEST score was >12, the sensitivity was 91.3%, and the specificity was 77.4%. We found that the area under the curve of the PRIEST score (0.887, *p* < 0.05) for mortality prediction was greater than that of the quick Sequential Organ Failure Assessment (0.724), CRB-65 (0.780), Rapid Emergency Medicine Score (0.809), and National Early Warning Score 2 (0.838). Moreover, prehospital PRIEST scores were positively correlated with numbers of comorbidities and numbers of prehospital treatment measures. The 30-day survival rate of patients with PRIEST scores ≤12 (98.8%) significantly exceeded that of patients with PRIEST scores >12 (69.1%) (*p* < 0.001). Prehospital PRIEST scores >12 (HR = 7.409) was one of the independent predictors of the 30-day mortality.

**Conclusions:**

The PRIEST can accurately, quickly, and conveniently predict the 30-day mortality of COVID-19 patients with pneumonia in the prehospital phase and can guide their initial risk stratification and treatment.

## Introduction

The clinical spectrum of coronavirus disease 2019 (COVID-19) ranges from asymptomatic infection to mild illness, moderate illness, severe illness, critical illness, and even death [1]. Compared with asymptomatic and mild patients, patients with pneumonia especially severely and critically ill patients have more comorbidities and complications. These patients also have more severe clinical signs and symptoms, require greater prehospital care workload and hospital medical resources, and have poorer outcomes [2–4]. A systematic review revealed that the pooled mean death rates of severely and critically ill patients (0.11) and those with pneumonia (0.03) were significantly greater than those of mildly ill patients without pneumonia (0.01) [2]. Moreover, COVID-19 patients with pneumonia suffer more severe damage to their health-related quality of life in the short and long term than those without pneumonia [5].

Due to the weak social healthcare system of developing countries, they still face the risk of losing control during an epidemic. Once a major outbreak of COVID-19 occurs, the demand for prehospital disposal, transport, inpatient treatment, and medical equipment may significantly increase because of the rapidly increasing number of patients in the short term [6,7]. This situation leads to a serious increase in medical resources, which may eventually lead to a sharp increase in patient mortality. Early identification, awareness, and intervention in patients with possibly fatal conditions may provide them with timely access to tailored treatment strategies and even alter outcomes.

Risk factors and models based on laboratory indicators such as high-sensitivity C-reactive protein (CRP), D-dimer, inflammatory cells, interleukins, plasma cell-free deoxyribonucleic acid (cfDNA), blood urea nitrogen (BUN), and lactic acid have been proven to accurately predict patient mortality [8–10]. In the first wave of COVID-19 and subsequent outbreaks, the physiological parameters scores represented by the National Early Warning Score 2 (NEWS2) and Pandemic Respiratory Infection Emergency System Triage (PRIEST) (as a modification of the NEWS2) have also demonstrated good performance in predicting the prognosis and risk stratification of suspected or confirmed patients [11,12]. However, these current studies still have some shortcomings that prevent their results from being extensively applied. First, some studies have targeted suspected patients rather than confirmed COVID-19 patients with pneumonia. Second, some studies defined prognosis as adverse events, not just death events. Third, some predictive models are complex to use due to the presence of laboratory indicators. Fourth, some physiological parameters scores use recommended fixed threshold values, resulting in poor specificity and a greater number of false ­positives. Finally, more studies have focused on the in-hospital phase rather than the prehospital phase, which may lead to delays in early warning.

Moreover, whether prehospital PRIEST can quickly, accurately, and economically predict the prognosis of COVID-19 patients with pneumonia has not been explored well. To fill this gap, the present study aimed to evaluate the performance of prehospital PRIEST in predicting the 30-day mortality of COVID-19 patients with pneumonia and to explore the relationship between prehospital PRIEST and condition and prehospital care.

## Materials and methods

### Study population

**Figure 1.**
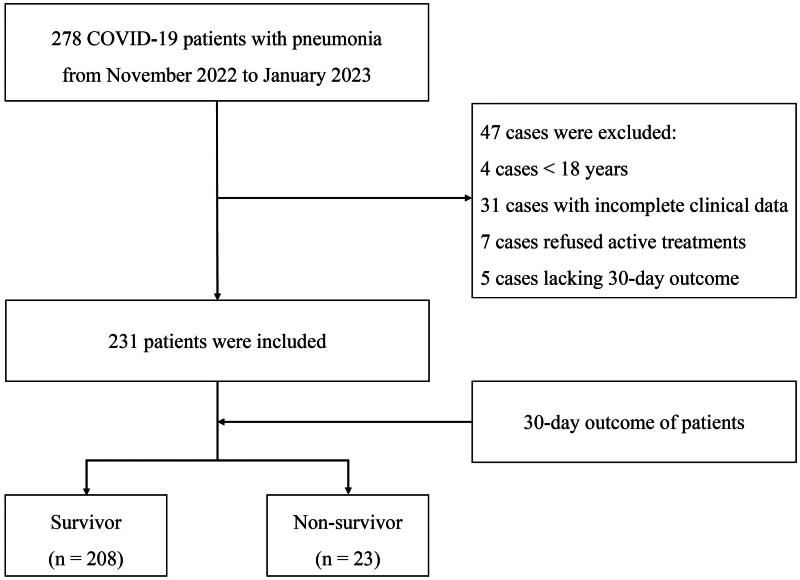
The flow chat of outline of the screening and case selection protocol. COVID-19, Coronavirus disease 2019.

This retrospective study included COVID-19 patients with pneumonia who were transferred to the Fifth Medical Center of PLA General Hospital which is a specialized institution dedicated to infectious diseases through prehospital care from November 2022 to January 2023. All of these patients voluntarily referred themselves for treatment at our institution. The inclusion criterion was the diagnosis of moderate, severe, or critical COVID-19 in accordance with the guidelines of National Institutes of Health (NIH) [1]. The exclusion criteria were age <18 years, incomplete clinical data, refusal of mechanical ventilation and other active treatments, and lack of 30-day outcome after admission. A total of 278 patients met the inclusion criteria, whereas 47 patients were excluded. 4 cases of age <18 years, 31 cases with incomplete clinical data, 7 cases who refused active treatments, and 5 cases lacking 30-day outcome after admission were excluded. Finally, 231 patients were selected for this study (Figure 1).

### Prehospital and in-hospital disposal

These patients were treated at fever clinics or emergency departments of other hospitals in Beijing, China. Patients were promptly transferred to our institution after being diagnosed with COVID-19 in other hospitals. During this period, patients received basic support treatment in other hospitals. According to their conditions, patients received prehospital care (oxygen therapy, transfusion, electrocardiogram (ECG) monitoring, mechanical ventilation, and stretcher or wheelchair handling) during the transport period. The patients were treated in the regular ward or intensive care unit (ICU) after transport. Patients received comprehensive treatment during hospitalization, including oxygen therapy, antiviral therapy (nirmatrelvir-ritonavir or azvudine), corticosteroids (methylprednisolone, dexamethasone or prednisone), and symptomatic support. According to illness severity, some patients received antibiotics, anticoagulant therapy, mechanical ventilation, and antishock treatment.

### Study design

We considered whether the patients died within 30 days after admission as the primary clinical outcome. When these patients left the fever clinic or emergency department of other hospitals, we defined it as the screening timepoint. The five physiological parameters scores of all patients at the beginning of prehospital transport were calculated. We collected the prehospital clinical data, prehospital transport parameters, and five prehospital physiological parameters scores of all patients. Receiver operating characteristic (ROC) curves and decision curve analysis were utilized to evaluate the accuracy of the five prehospital physiological parameter scores for predicting the 30-day mortality. Based on the threshold value of the best performer, these patients were categorized into low-score and high-score groups. A comparison was made between the two groups regarding their basic condition, prehospital care workload, and the 30-day mortality. Patients were categorized into survival and non-survival groups based on the 30-day mortality. The clinical characteristics of these two groups were then compared, and the independent predictors of the 30-day mortality were identified.

### Definitions

COVID-19 patients are diagnosed by positive severe acute respiratory syndrome coronavirus 2 reverse-transcription polymerase chain reaction tests *via* nasopharyngeal swabs and have any of the various signs and symptoms [1]. The clinical spectrum of COVID-19 is defined by the guidelines of NIH. Moderate state is defined as lower respiratory disease and SpO_2_ ≥94%. Severe state is defined as SpO_2_ <94%, a ratio of arterial partial pressure of oxygen to fraction of inspired oxygen <300 mmHg, a respiratory rate >30 breaths/min, or lung infiltrates >50%. Critical state is defined as respiratory failure, septic shock, and/or multiple organ dysfunction [1]. All COVID-19 patients with pneumonia were diagnosed after discussion among the members of our institution expert group. Septic shock is defined by the Sepsis 3.0 criteria [13]. The Quick Sequential Organ Failure Assessment (qSOFA) comprises the three physiological parameters of altered mentation, respiratory rate (RR), and systolic blood pressure (SBP), providing an overall score between 0 and 3 [13]. The Rapid Emergency Medicine Score (REMS) consists of the six physiological parameters of pulse rate (PR), mean arterial pressure (MAP), RR, Glasgow Coma Scale (GCS), age and oxygen saturation (SpO_2_), providing a total score between 0 and 26 [14]. The CRB-65 comprises the four physiological parameters of confusion, RR, blood pressure, and age ≥65 years old, to give an overall score between 0 and 4 [15]. The NEWS2 can be calculated using the seven physiological parameters of RR, SpO_2_, PR, SBP, temperature, consciousness, and oxygen therapy, providing a total score between 0 and 20 [16]. On the basis of the NEWS2, three physiological parameters, age, sex, and performance status, were added to the PRIEST to obtain an overall score between 0 and 29 [12].

### Statistical analysis

Categorical variables were described as percentages (%), and continuous variables were expressed as the mean ± SD or median (interquartile range, P25–P75). Continuous variables with a normal distribution were compared by conducting independent-samples t-test. The Mann–Whitney *U* test was performed to compare continuous variables with a nonnormal distribution. Categorical variables were compared by the chi-square test, continuous correction chi-square test, or Fisher’s exact test, as appropriate. Correlations between two groups of variables were calculated using the Spearman method. The sensitivity, specificity, positive predictive value (PPV), and negative predictive value (NPV) of the five physiological parameters scores were calculated by ROC curves. The area under the curve (AUC) of the two physiological parameters scores were compared by DeLong’s method. Cumulative survival probability curves were calculated with the Kaplan–Meier method and compared through the log-rank test. The occurrence of death within 30 days after admission was considered the dependent variable. Independent predictors were analyzed to discriminate patient death by Cox proportional hazard regression. Candidate variables (*p* < 0.05) were used as inputs for multivariable COX proportional hazard regression analysis following a forward stepwise approach (p-in: 0.05, p-out: 0.10). The calibration curve, decision curve analysis (DCA), and clinical impact curve (CIC) were used to evaluate the predictive ability of the PRIEST score. Statistical analyses and mapping were conducted using SPSS 23.0 (IBM, Armonk, NY) and R version 4.3.1 (http://www.r-project.org). *p* < 0.05 was considered to indicate statistical significance in the analyses.

## Results

### Baseline clinical characteristics of all patients

This study included 231 patients with COVID-19, including 139 with moderate cases, 74 with severe cases, and 18 with critical cases, with an average age of 81.0 (53.0–93.0) years. Approximately 71.4% of the patients were male, and the number of days from onset of symptom to admission was 3.0 (1.0–7.0). Among the patients, 78.8% had comorbidities, with the common comorbidities being hypertension (51.9%), chronic cardiac disease (47.6%), and diabetes (27.3%). Fever (88.3%), cough (77.1%), and fatigue (39.4%) were the common signs and symptoms. Approximately 3.0% of patients were complicated by septic shock. Moreover, 48.9%, 31.2%, 22.1%, and 5.2% of the patients received oxygen therapy, ECG monitoring, transfusion, and mechanical ventilation, respectively. Among them, 56.7% used stretchers or wheelchairs during the prehospital transport phase. The qSOFA, REMS, CRB-65, NEWS2, and PRIEST scores of the patients were 0.0 (0.0–0.0), 6.0 (3.0–8.0), 1.0 (0.0–1.0), 2.0 (0.0–5.0), and 9.0 (3.0–13.0), respectively. Among the patients, 62.3% and 29.0% of patients received antiviral therapy and corticosteroids during hospitalization, respectively (Table 1).

**Table 1.** Clinical characteristics of COVID-19 patients with pneumonia.

Variables	All patients (*n* = 231)	Non-survivor group (*n* = 23)	Survivor group (*n* = 208)	*P*
Age (years)	81.0 (53.0–93.0)	93.0 (86.0–94.0)	78.0 (49.5–92.0)	<0.001
Male, n (%)	165 (71.4)	20 (87.0)	145 (69.7)	0.082
Unvaccinated, n (%)	78 (33.8)	11 (47.8)	67 (32.2)	0.180
Onset of symptom to admission (days)	3.0 (1.0–7.0)	6.0 (3.0–9.0)	3.0 (1.0–7.0)	0.002
**Comorbidities, n (%)**				
Hypertension	120 (51.9)	19 (82.6)	101 (48.6)	0.002
Diabetes	63 (27.3)	8 (34.8)	55 (26.4)	0.394
Chronic cardiac disease	110 (47.6)	15 (65.2)	95 (45.7)	0.075
Chronic pulmonary disease	26 (11.3)	6 (26.1)	20 (9.6)	0.043
Chronic kidney disease	47 (20.3)	9 (39.1)	38 (18.3)	0.037
Cerebrovascular disease	54 (23.4)	8 (34.8)	46 (22.1)	0.173
Chronic liver disease	16 (6.9)	2 (8.7)	14 (6.7)	1.000
Active malignancy	31 (13.4)	4 (17.4)	27 (13.0)	0.524
Numbers of comorbidities	2.0 (1.0–3.0)	3.1 ± 1.1	2.0 (1.0–3.0)	<0.001
**Signs and symptoms, n (%)**				
Fever	204 (88.3)	22 (95.7)	182 (87.5)	0.416
Cough	178 (77.1)	22 (95.7)	156 (75.0)	0.025
Sore throat	67 (29.0)	1 (4.3)	66 (31.7)	0.006
Nasal obstruction	12 (5.2)	0 (0.0)	12 (5.8)	0.491
Fatigue	91 (39.4)	20 (87.0)	71 (34.1)	<0.001
Headache	12 (5.2)	0 (0.0)	12 (5.8)	0.491
Myalgia	35 (15.2)	0 (0.0)	35 (16.8)	0.067
Anorexia	62 (26.8)	17 (73.9)	45 (21.6)	<0.001
Diarrhea	6 (2.6)	1 (4.3)	5 (2.4)	0.471
Dyspnea	48 (20.8)	15 (65.2)	33 (15.9)	<0.001
Anosmia	6 (2.6)	0 (0.0)	6 (2.9)	1.000
Numbers of signs and symptoms	3.0 (2.0–4.0)	4.0 (4.0–5.0)	3.0 (2.0–4.0)	<0.001
**Prehospital parameters**				
SpO_2_ (%)	96.0 (93.0–98.0)	92.0 (88.0–93.0)	96.0 (95.0–98.0)	<0.001
Temperature (°C)	36.8 (36.5–37.4)	36.7 (36.5–37.6)	36.8 (36.5–37.4)	0.755
RR (breaths/min)	19.0 (18.0–20.0)	20.0 (18.0–22.0)	19.0 (18.0–20.0)	0.007
PR (beats/minute)	78.0 (74.0–89.0)	79.0 (72.0–96.0)	78.0 (74.0–88.0)	0.959
SBP (mmHg)	132.0 (122.0–141.0)	133.8 ± 26.1	131.0 (122.0–140.0)	0.515
DBP (mmHg)	79.1 ± 10.5	76.4 ± 13.7	79.4 ± 10.0	0.319
MAP (mmHg)	96.7 (90.0–103.7)	95.5 ± 14.8	96.7 (90.3–103.3)	0.568
GCS	15.0 (15.0–15.0)	14.0 (12.0–15.0)	15.0 (15.0–15.0)	<0.001
**Complications, n (%)**				
Septic shock	7 (3.0)	4 (17.4)	3 (1.4)	0.002
**Prehospital care**				
Oxygen therapy, n (%)	113 (48.9)	22 (95.7)	91 (43.8)	<0.001
Transfusion, n (%)	51 (22.1)	15 (65.2)	36 (17.3)	<0.001
ECG monitoring, n (%)	72 (31.2)	19 (82.6)	53 (25.5)	<0.001
Mechanical ventilation, n (%)	12 (5.2)	7 (30.4)	5 (2.4)	<0.001
Use of stretcher or wheelchair, n (%)	131 (56.7)	23 (100.0)	108 (51.9)	<0.001
Numbers of treatment measures	1.0 (0.0–3.0)	4.0 (3.0–5.0)	1.0 (0.0–2.8)	<0.001
**Physiological parameters scores**				
qSOFA	0.0 (0.0–0.0)	1.0 (0.0–2.0)	0.0 (0.0–0.0)	<0.001
REMS	6.0 (3.0–8.0)	8.0 (7.0–11.0)	6.0 (2.0–7.8)	<0.001
CRB-65	1.0 (0.0–1.0)	2.0 (1.0–2.0)	1.0 (0.0–1.0)	<0.001
NEWS2	2.0 (0.0–5.0)	7.4 ± 3.7	2.0 (0.0–5.0)	<0.001
PRIEST	9.0 (3.0–13.0)	15.9 ± 3.6	8.0 (3.0–12.0)	<0.001
**Treatment after admission, n (%)**				
Antiviral therapy	144 (62.3)	11 (47.8)	133 (63.9)	0.130
Corticosteroids	67 (29.0)	17 (73.9)	50 (24.0)	<0.001

COVID-19, Coronavirus disease 2019; SpO_2,_ Oxygen saturation; RR, Respiratory rate; PR, Pulse rate; SBP, Systolic blood pressure; DBP, Diastolic blood pressure; MAP, Mean arterial pressure; GCS, Glasgow coma scale; ECG, Electrocardiogram; qSOFA, quick Sequential Organ Failure Assessment; REMS, Rapid Emergency Medicine Score; NEWS2, National Early Warning Score 2; PRIEST, Pandemic Respiratory Infection Emergency System Triage.

Categorical variables were expressed as n (%), and continuous variables were described as mean ± SD or median (interquartile range, P25–P75) depending on whether they followed normal distribution.

*P*: Comparison between survivor and non-survivor groups.

### Clinical characteristics of survivor and non-survivor groups

According to the 30-day outcome, all patients were divided into survivor (208 cases) and non-survivor (23 cases) groups. The age, onset of symptom to admission, hypertension, chronic pulmonary disease, chronic kidney disease, numbers of comorbidities, cough, fatigue, anorexia, dyspnea, numbers of signs and symptoms, RR, septic shock, prehospital care, corticosteroid therapy, and five physiological ­parameters scores of the non-survivor group were higher than those of the survivor group (*p* < 0.05) (Table 1). In contrast, the sore throat, SpO_2_, and GCS scores of the non-survivor group were lower than those of the survivor group (*p* < 0.05) (Table 1).

### Performance of prehospital PRIEST scores in predicting the 30-day mortality

**Figure 2.**
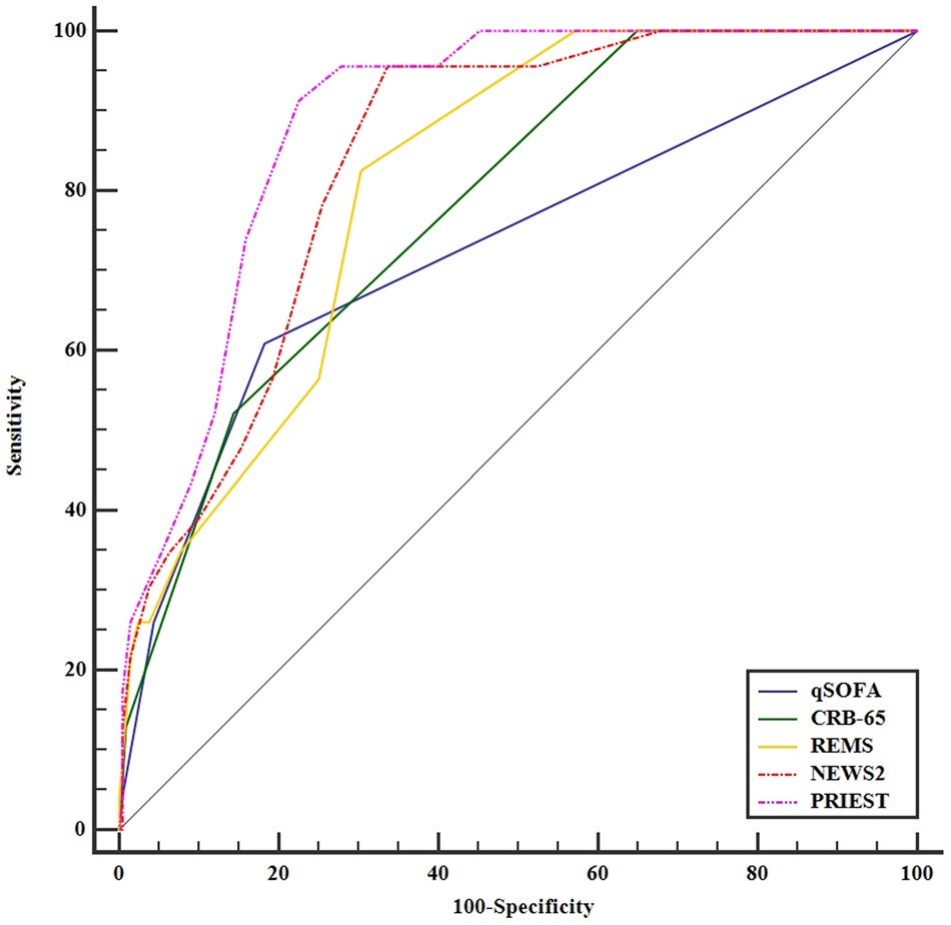
The receiver operating characteristic curves of the five prehospital physiological parameters scores for discriminate the 30-day outcome.

**Figure 3.**
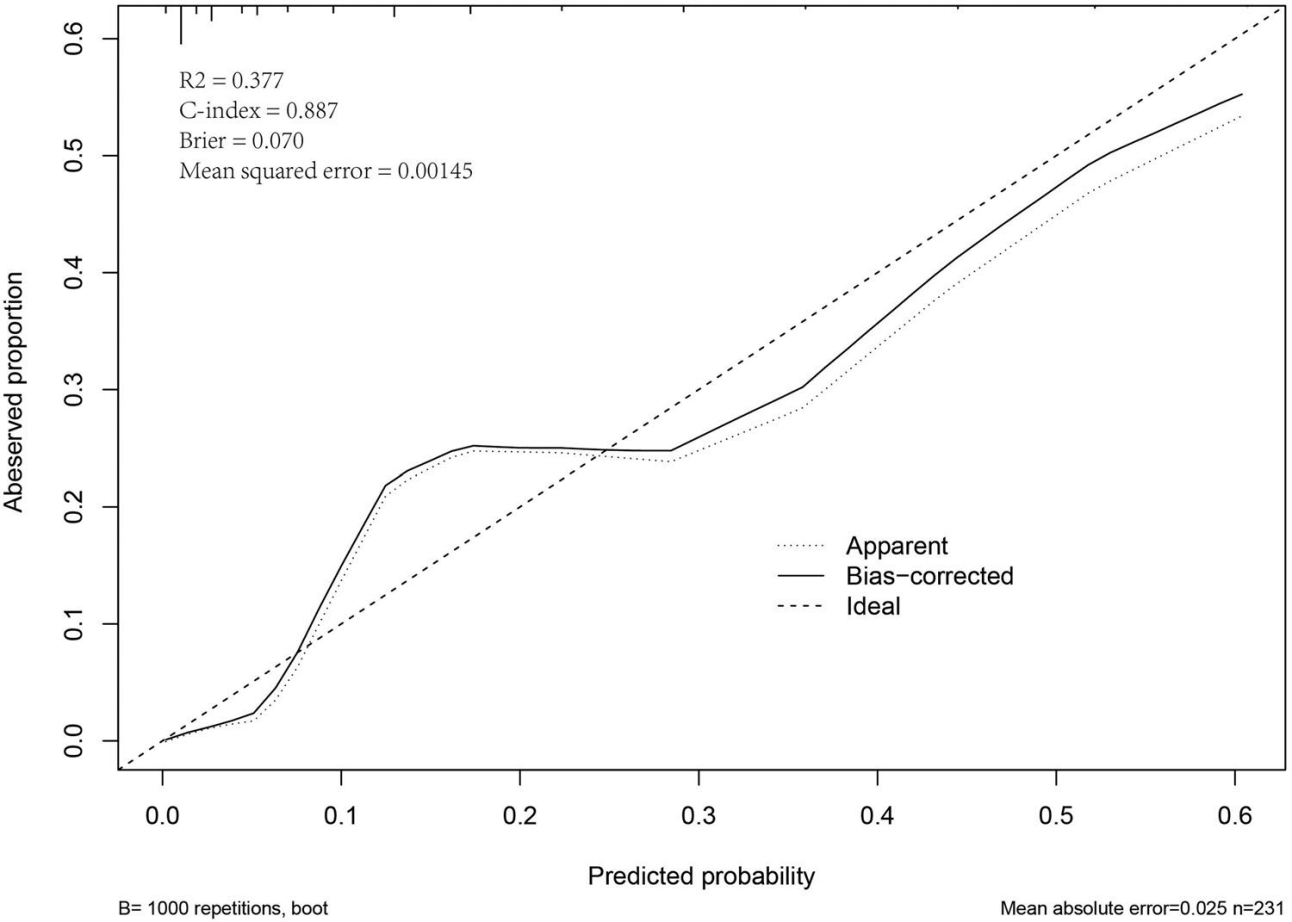
The calibration curve to assess the calibration of the prehospital PRIEST scores by the bootstrap. PRIEST, Pandemic Respiratory Infection Emergency System Triage.

**Figure 4.**
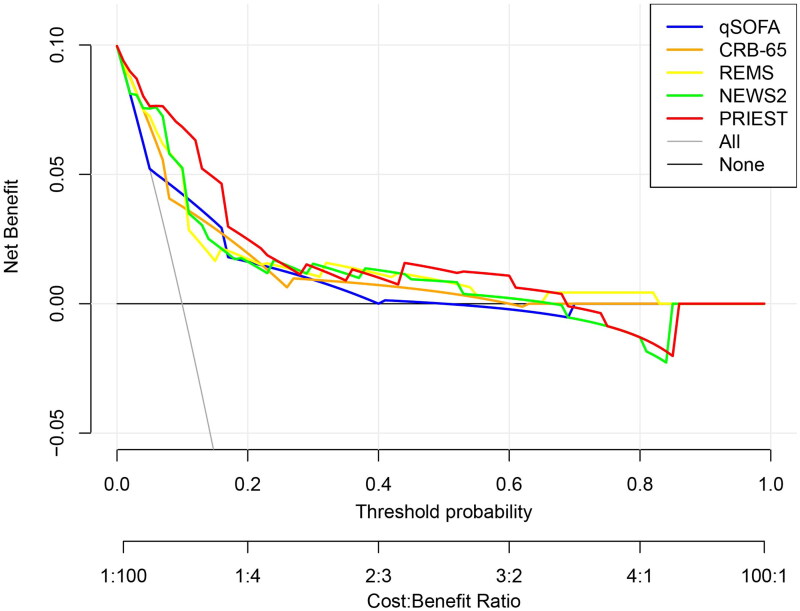
The decision curve analysis curve of the prediction of the mortality risk with the five prehospital physiological parameters scores.

**Figure 5.**
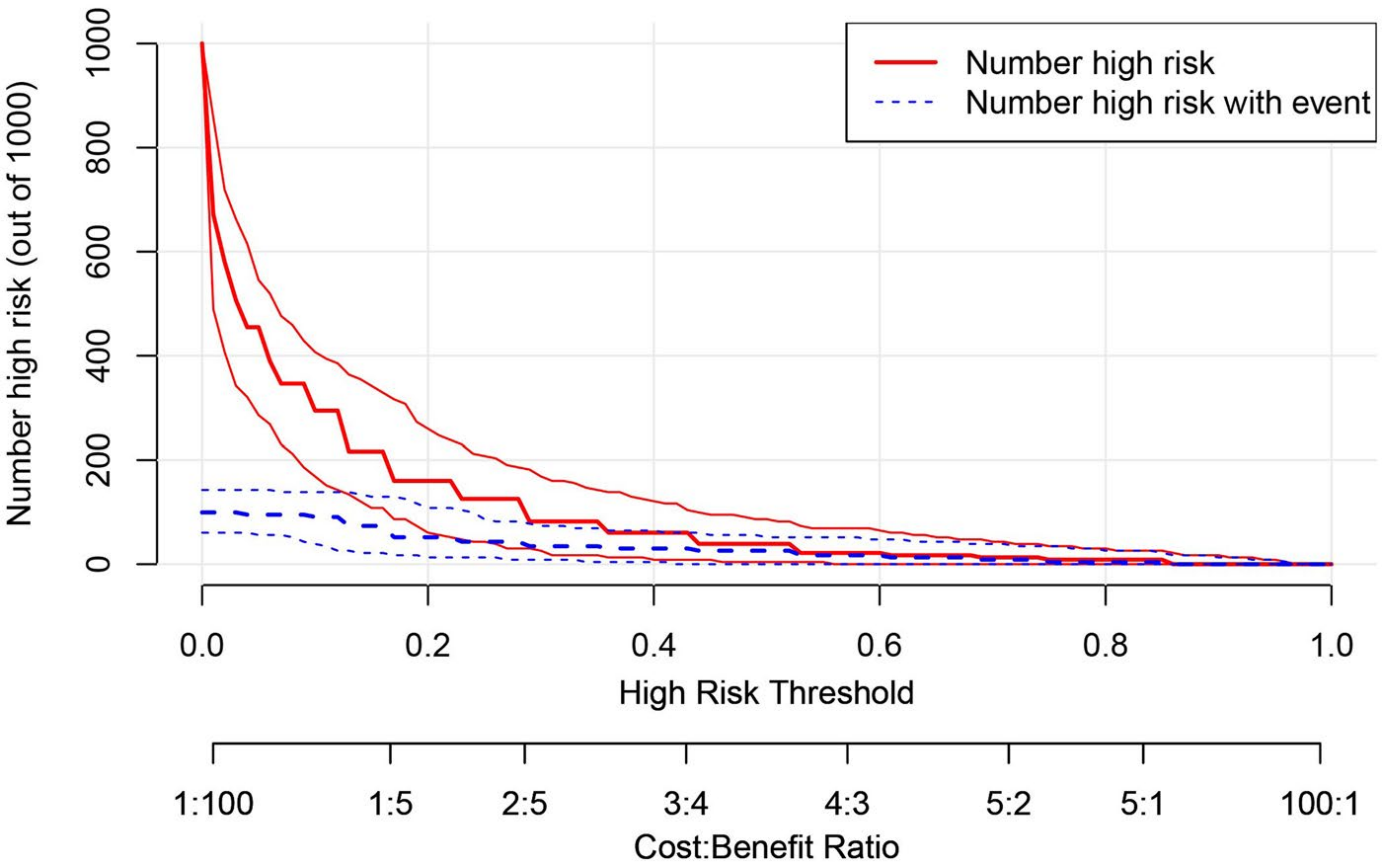
The clinical impact curve for the prediction of the mortality risk with the prehospital PRIEST scores. PRIEST, Pandemic Respiratory Infection Emergency System Triage.

The AUC, threshold, Youden index, sensitivity, specificity, PPV, and NPV of prehospital PRIEST scores were 0.887 (95% CI, 0.839–0.925), >12, 0.687, 91.3 (95% CI, 72.0–98.9)%, 77.4 (95% CI, 71.1–82.9)%, 30.9 (95% CI, 20.2–43.3)%, and 98.8 (95% CI, 95.6–99.9)%, respectively (Figure 2). The AUCs of the prehospital PRIEST score (0.887) were much greater than those of the qSOFA score (0.724, *p* < 0.001), the CRB-65 score (0.780, *p* < 0.001), the REMS score (0.809, *p* = 0.034), and the NEWS2 score (0.838, *p* < 0.001) (Table 2). The Hosmer–Lemeshow goodness-of-fit test indicated that the PRIEST had good calibration (*p* = 0.443). The Brier score was 0.070 according to the calibration curve, which was close to 0, indicating that the prehospital PRIEST scores showed good consistency between the predicted 30-day mortality and the actual 30-day mortality, although the bias-corrected curve showed a slight deviation from the reference line (Figure 3). The DCA curve showed that when the predicted threshold probability was between 3% and 70%, the use of the prehospital PRIEST scores to make clinical decisions achieved greater net benefits than did the use of the other four prehospital physiological parameters scores (Figure 4). CIC analysis revealed that when the threshold probability was above 45%, the number of subjects who were at a high risk of predicted positive cases was highly matched with the number of subjects who were at a high risk of true positive cases, confirming the good clinical effectiveness of prehospital PRIEST scores (Figure 5).

**Table 2.** Performance of the five prehospital physiological parameters scores in predicting the 30-day mortality.

Physiological parameters scores	AUC (95% CI)	Threshold	Youden indices	Sensitivity (%) (95% CI)	Specificity (%) (95% CI)	PPV (%) (95% CI)	NPV (%) (95% CI)	*P*
qSOFA	0.724 (0.662–0.781)	>0	0.426	60.9 (38.5–80.3)	81.7 (75.8–86.7)	26.9 (15.6–41.0)	95.0 (90.7–97.7)	<0.001
CRB-65	0.780 (0.721–0.831)	>1	0.378	52.2 (30.6–73.2)	85.6 (80.1–90.1)	28.6 (15.7–44.6)	94.2 (89.8–97.1)	<0.001
REMS	0.809 (0.752–0.858)	>6	0.523	82.6 (61.2–95.0)	69.7 (63.0–75.9)	23.2 (14.6–33.8)	97.3 (93.3–99.3)	0.034
NEWS2	0.838 (0.784–0.883)	>3	0.620	95.7 (78.1–99.9)	66.4 (59.5–72.7)	23.9 (15.6–33.9)	99.3 (96.1–100.0)	<0.001
PRIEST	0.887 (0.839–0.925)	>12	0.687	91.3 (72.0–98.9)	77.4 (71.1–82.9)	30.9 (20.2–43.3)	98.8 (95.6–99.9)	/

AUC, area under the curve; PPV, positive predictive value; NPV, negative predictive value; qSOFA, quick Sequential Organ Failure Assessment; REMS, Rapid Emergency Medicine Score; NEWS2, National Early Warning Score 2; PRIEST, Pandemic Respiratory Infection Emergency System Triage.

*P*: Comparison of other physiological parameters scores with PRIEST scores.

### Relationship of prehospital PRIEST scores with patient’s condition and prehospital care

Prehospital PRIEST scores were positively correlated with the numbers of comorbidities (*r* = 0.654, *p* < 0.001), numbers of signs and symptoms (*r* = 0.313, *p* < 0.001), ­numbers of prehospital treatment measures (*r* = 0.871, *p* < 0.001), and days of onset of symptom to admission (*r* = 0.392, *p* < 0.001). The numbers of comorbidities, numbers of signs and symptoms, numbers of prehospital treatment measures, and days from onset of symptom to admission of patients with PRIEST scores >12 were all greater than those of patients with PRIEST scores ≤12 (*p* < 0.001). The age, unvaccinated, RR, and septic shock of the PRIEST scores >12 group were higher than those of the PRIEST scores ≤12 group (*p* < 0.05). In contrast, the SpO_2_, and GCS scores of the PRIEST scores >12 group were lower than those of the PRIEST scores ≤12 group (*p* < 0.001) (Table 3).

**Table 3.** Comparisons of patient’s condition and prehospital care between the prehospital PRIEST scores >12 and PRIEST scores ≤12.

Variables	PRIEST scores >12 (*n* = 68)	PRIEST scores ≤12 (*n* = 163)	*P*
Age (years)	92.0 (84.0–94.0)	67.0 (45.0–89.0)	<0.001
Male, n (%)	54 (79.4)	111 (68.1)	0.083
Unvaccinated, n (%)	33 (48.5)	45 (27.6)	0.002
Onset of symptom to admission (days)	5.5 (2.3–9.0)	2.0 (1.0–6.0)	<0.001
**Comorbidities, n (%)**			
Hypertension	45 (66.2)	75 (46.0)	0.005
Diabetes	22 (32.4)	41 (25.2)	0.263
Chronic cardiac disease	40 (58.8)	70 (42.9)	0.028
Chronic pulmonary disease	15 (22.1)	11 (6.7)	0.001
Chronic kidney disease	21 (30.9)	26 (16.0)	0.010
Cerebrovascular disease	34 (50.0)	20 (12.3)	<0.001
Chronic liver disease	6 (8.8)	10 (6.1)	0.653
Active malignancy	9 (13.2)	22 (13.5)	0.958
Numbers of comorbidities	3.0 (2.0–4.0)	2.0 (0.0–3.0)	<0.001
**Signs and symptoms, n (%)**			
Fever	66 (97.1)	138 (84.7)	0.008
Cough	61 (89.7)	117 (71.8)	0.003
Sore throat	10 (14.7)	57 (35.0)	0.002
Nasal obstruction	2 (2.9)	10 (6.1)	0.502
Fatigue	40 (58.8)	51 (31.3)	<0.001
Headache	1 (1.5)	11 (6.7)	0.186
Myalgia	6 (8.8)	29 (17.8)	0.083
Anorexia	33 (48.5)	29 (17.8)	<0.001
Diarrhea	4 (5.9)	2 (1.2)	0.116
Dyspnea	29 (42.6)	19 (11.7)	<0.001
Anosmia	4 (5.9)	2 (1.2)	0.116
Numbers of signs and symptoms	4.0 (3.0–5.0)	3.0 (2.0–4.0)	<0.001
**Prehospital parameters**			
SpO_2_ (%)	93.0 (91.0–95.0)	97.0 (95.0–98.0)	<0.001
Temperature (°C)	36.8 (36.5–37.6)	36.8 (36.4–37.2)	0.117
RR (breaths/min)	20.0 (19.0–22.0)	18.0 (18.0–19.0)	<0.001
PR (beats/minute)	81.5 (72.0–99.8)	78.0 (74.0–86.0)	0.102
SBP (mmHg)	131.4 ± 24.7	131.0 (122.0–141.0)	0.860
DBP (mmHg)	76.8 ± 11.7	79.0 (74.0–87.0)	0.057
MAP (mmHg)	95.0 ± 13.2	96.7 (90.3–104.0)	0.135
GCS	13.0 (9.0–15.0)	15.0 (15.0–15.0)	<0.001
**Complications, n (%)**			
Septic shock	7 (10.3)	0 (0.0)	<0.001
**Prehospital care**			
Oxygen therapy, n (%)	64 (94.1)	49 (30.1)	<0.001
Transfusion, n (%)	38 (55.9)	13 (8.0)	<0.001
ECG monitoring, n (%)	52 (76.5)	20 (12.3)	<0.001
Mechanical ventilation, n (%)	11 (16.2)	1 (0.6)	<0.001
Use of stretcher or wheelchair, n (%)	68 (100.0)	63 (38.7)	<0.001
Numbers of treatment measures	4.0 (3.0–4.0)	0.0 (0.0–1.0)	<0.001
**Physiological parameters scores**			
qSOFA	1.0 (0.0–1.0)	0.0 (0.0–0.0)	<0.001
REMS	8.0 (6.3–9.0)	5.0 (2.0–6.0)	<0.001
CRB-65	2.0 (1.0–2.0)	1.0 (0.0–1.0)	<0.001
NEWS2	7.0 (5.0–9.0)	1.0 (0.0–3.0)	<0.001
PRIEST	15.0 (13.0–17.0)	6.0 (2.0–9.0)	<0.001

COVID-19, Coronavirus disease 2019; PRIEST, Pandemic Respiratory Infection Emergency System Triage; SpO_2,_ Oxygen saturation; RR, Respiratory rate; PR, Pulse rate; SBP, Systolic blood pressure; DBP, Diastolic blood pressure; MAP, Mean arterial pressure; GCS, Glasgow coma scale; ECG, Electrocardiogram; qSOFA, quick Sequential Organ Failure Assessment; REMS, Rapid Emergency Medicine Score; NEWS2, National Early Warning Score 2.

Categorical variables were expressed as n (%), and continuous variables were described as mean ± SD or median (interquartile range, P25–P75) depending on whether they followed normal distribution.

*P*: Comparison between the PRIEST scores >12 and PRIEST scores ≤12.

### The 30-day mortality of patients

**Figure 6.**
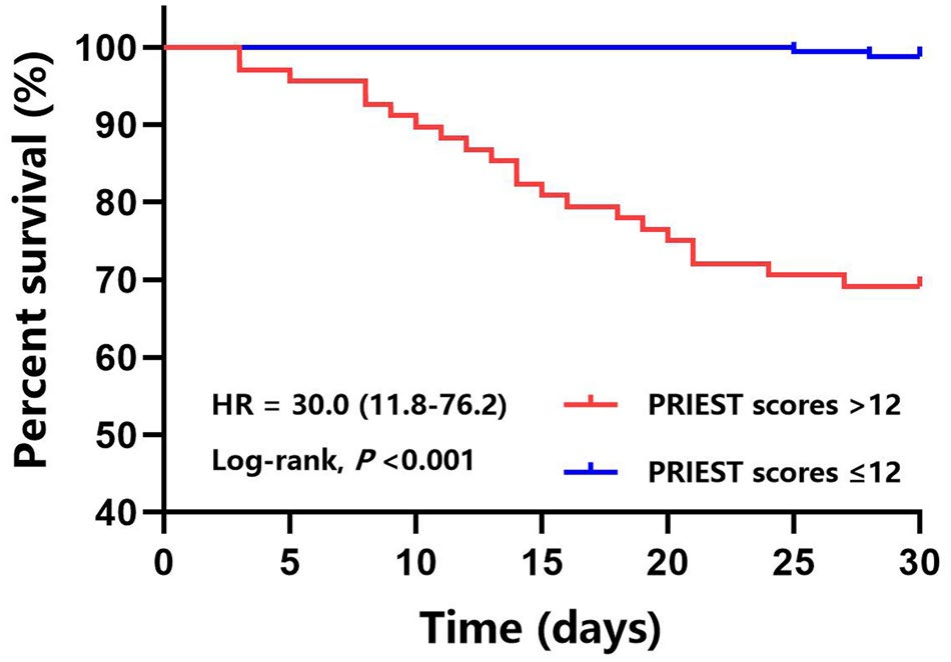
Comparison of the 30-day survival rates between the prehospital PRIEST scores >12 group and PRIEST scores ≤12 group. PRIEST, Pandemic Respiratory Infection Emergency System Triage.

On the 30th day after admission, the overall mortality of all patients was 10.0%. Twenty-three patients died at 15.0 ± 7.4 days after admission. Results of Kaplan–Meier analysis indicated that the 30-day survival rates in the prehospital PRIEST scores ≤12 group (98.8%) were significantly greater than those in the prehospital PRIEST scores >12 group (69.1%) (*p* < 0.001) (Figure 6).

### Independent predictors of the 30-day mortality

Patients who died within 30 days after admission served as the dependent variable in the univariable COX proportional hazard regression. To avoid multicollinearity, in the univariate COX proportional hazard regression, we excluded the nine independent variables that made up the PRIEST score, including RR, SpO_2_, oxygen therapy, temperature, SBP, PR, GCS, age, and sex. The independent variables were comorbidities, signs and symptoms, prehospital parameters, complications, unvaccinated, onset of symptom to admission, prehospital care, and prehospital PRIEST scores >12. Hypertension, chronic pulmonary disease, chronic kidney disease, sore throat, fatigue, anorexia, dyspnea, septic shock, onset of symptom to admission, transfusion, ECG monitoring, mechanical ventilation, use of stretcher or wheelchair, and prehospital PRIEST scores >12 were screened out as meaningful variables. These variables served as inputs for multivariate Cox proportional hazard regression (*p* < 0.05) (Table 4). Multivariate Cox proportional hazard regression showed that hypertension (HR =3.496), chronic pulmonary disease (HR =3.591), fatigue (HR =10.537), septic shock (HR =4.041), and prehospital PRIEST scores >12 (HR =14.292) were independent predictors of the 30-day mortality of the patients (*p* < 0.05) (Table 4).

**Table 4.** Independent predictors for the 30-day mortality in COVID-19 patients with pneumonia.

Variables	Univariate Cox regression		Multivariate Cox regression	
	HR (95% CI)	*P*	Adjusted HR (95% CI)	*P*
Hypertension	4.690 (1.595–13.787)	0.005	3.496 (1.144–10.682)	0.028
Chronic pulmonary disease	3.078 (1.213–7.809)	0.018	3.591 (1.288–10.010)	0.015
Chronic kidney disease	2.621 (1.134–6.057)	0.024		
Sore throat	0.106 (0.014–0.783)	0.028		
Fatigue	11.455 (3.403–38.564)	<0.001	10.537 (2.979–37.272)	<0.001
Anorexia	8.755 (3.449–22.224)	<0.001		
Dyspnea	8.094 (3.429–19.108)	<0.001		
Septic shock	9.789 (3.318–28.874)	<0.001	4.041 (1.258–12.985)	0.019
Onset of symptom to admission	1.153 (1.058–1.257)	0.001		
Transfusion	7.939 (3.363–18.741)	<0.001		
ECG monitoring	12.241 (4.162–36.005)	<0.001		
Mechanical ventilation	12.201 (4.994–29.809)	<0.001		
Use of stretcher or wheelchair	55.232 (1.890–1613.670)	0.02		
PRIEST scores >12	30.236 (7.084–129.048)	<0.001	14.292 (3.254–62.776)	<0.001

COVID-19, Coronavirus disease 2019; HR, Hazard ratio; ECG, Electrocardiogram; PRIEST, Pandemic Respiratory Infection Emergency System Triage.

## Discussion

This study on the performance of prehospital PRIEST scores for predicting the 30-day mortality of COVID-19 patients with pneumonia revealed three main findings. First, compared with survivor patients, non-survivor ones were significantly older and had greater numbers of comorbidities, signs and symptoms, complications, and physiological parameters scores. They also required greater prehospital care. Second, prehospital PRIEST scores >12 was selected as the threshold value, it was the best performer in effectively predicting the 30-day mortality of COVID-19 patients with pneumonia and was also an important independent predictor for their short-term prognosis. Third, prehospital PRIEST scores were positively correlated with the numbers of comorbidities, signs and symptoms, and prehospital treatment measures, and days from onset of symptom to admission.

Overall, compared with survivors of COVID-19 in the short term, non-survivors were older and had more chronic pulmonary and cardiac diseases, poorer consciousness, faster respiratory rate, and lower SpO_2_. Non-survivors had higher inflammatory indicators, proportion of mechanical ventilation, and vasoactive drug therapy than survivors [8,10,17,18]. Our research findings were consistent with previous ones. Notably, this study focused on prehospital phase and also found that non-survivors had longer days of onset of symptom to admission and a higher proportion of septic shock. The prehospital care workload and physiological parameters scores for non-survivors were significantly higher than those for survivors, and non-survivors consumed more prehospital service resources. Therefore, COVID-19 patients with pneumonia needed to conduct an accurate assessment for short-term prognosis as early as possible to optimize clinical management and medical-resource allocation. Early, rapid, and convenient screening and warning of patients with poor prognosis should have important clinical value. Pre-existent characteristics (age, sex, signs and ­symptoms, comorbidities, complications, etc.) [4,19,20], physiological parameters (SpO_2_, temperature, PR, consciousness disorders, etc.) [10,19], laboratory indicators (CRP, BUN, white blood cell count, lymphocyte count, D-dimer, platelets, etc.) [8,10,19,20], imaging data [21], viral variant and vaccination status [22,23], treatment methods [20,24], and the corresponding clinical models [8–10,19], as well as combinations of the above, are the most frequent predictive factors of mortality in patients with COVID-19. The current work also showed that pre-existent characteristics (fatigue, hypertension, chronic pulmonary disease, and septic shock) and physiological parameters (prehospital PRIEST) were the independent predictors of 30-day mortality. Septic shock is an important characteristic of critical state [1] that can predict the prognosis of patients with COVID-19 [4] and is also one of their common immediate causes of death [25]. Patients with septic shock need to receive vasopressor treatment and strict prehospital care, so sufficient medical human resources should be arranged during the transport period. Paying attention to fatigue or exhaustion as a common nonspecific symptom of COVID-19 is important because the disease itself produce this symptom and the progression of COVID-19 may also increase its severity [26,27]. One model that can accurately predict the prognosis of serious COVID-19 comprises ≥3 comorbidities, acute physiology and chronic health evaluation II (APACHE II) scores, BUN, lactic acid, the percentage of lymphocytes, and the neutrophil-to-platelet ratio. The AUC of this model for the training set is 0.880, and that for the validation set is 0.814 [8]. Age, tracheal intubation, shock status, plasma cfDNA, and BUN are screened as independent risk factors for death in serious COVID-19. The AUC of this nomogram model is 0.856, demonstrating a good predictive value of the 60-day outcome [9]. A model based on laboratory indicators and physiological parameters shows that CRP, D-dimer, and RR can simply predict the 30-day outcome of patients with COVID-19. The predictive effect of this model has been confirmed to be comparable with those of SOFA and CRUB-65 [10]. These models have good predictive value in the previous literature, but they have limited generalizability and applicability in developing countries due to their reliance on laboratory indicators that are not fast or economical.

Physiological parameters scoring is non-invasive, rapid, and cost-effective. Some studies have demonstrated the potential of physiological parameters scores in predicting COVID-19 mortality and identifying the severe/critical state. Consistent with this current research findings, previous studies have indicated that REMS, NEWS2, CRB-65, and qSOFA, which are frequently utilized in prehospital and emergency assessment of pneumonia, effectively predict the in-hospital mortality of patients with COVID-19 [28,29]. The NEWS2 (AUC =0.880), which is recommended by the Royal College of Physicians, has better predictive value than the REMS (AUC =0.839), the CRB-65 (AUC =0.766), and the qSOFA (AUC =0.694) in predicting the in-hospital death of patients with COVID-19, because it covers more physiological parameters closely related to the prognosis of this disease [10,11,19]. The prehospital NEWS2 (AUC =0.963) can also accurately and quickly distinguish severe/critical COVID-19 during the Omicron variant wave. NEWS2 scores >2 indicate an increase in prehospital care workload and consumption of medical human resources [6]. A study involving 70 emergency departments across the UK and including over 20,000 patients with suspected COVID-19 confirm that the PRIEST score formed by adding three physiological parameters (age, sex, and performance status) on the basis of the NEWS2 is more helpful to predict adverse outcome. A PRIEST score >4 (AUC =0.80, sensitivity =0.98, specificity =0.34) demonstrated effective discrimination in predicting death or organ support within 30 days in these patients. It can support decision making in emergency care [12]. For the same type of patients, literature from the UK [30] and South Africa [31] has relied on the fixed threshold value (PRIEST scores >4) from a prior study [12]. These studies indicate that the PRIEST exhibits satisfactory predictive efficacy for the 30-day mortality (AUC =0.83–0.85, sensitivity =0.85–0.98), but its specificity (0.39–0.64) is relatively low [30,31]. For confirmed patients, the PRIEST (AUC =0.846, sensitivity =0.875, specificity =0.711) and NEWS2 (AUC =0.843) indices offer superior predictive accuracy for the 30-day mortality. The predictive value of the PRIEST score is notably greater than that of the qSOFA score (AUC =0.788) when the PRIEST score is ≥8 [18]. A Greek study (AUC =0.852) on predicting adverse outcomes (death or need for invasive ventilation) of COVID-19 patients with pneumonia is consistent with the threshold value of PRIEST scores obtained from our current study [32]. The ability of prehospital PRIEST to rapidly discriminate patients with serious COVID-19 is comparable to that of the NEWS2 and surpasses that of the qSOFA, CRB-65, and REMS [6]. Our study further revealed that compared with the qSOFA, CRB-65, REMS, and NEWS2 scores, the prehospital PRIEST score can more accurately predict the 30-day mortality of COVID-19 patients with pneumonia. A clear positive correlation existed between prehospital PRIEST scores and patient’s condition and prehospital care workload. These findings from prehospital PRIEST may have significant implications for the clinical management of patients with COVID-19. First, the optimal threshold for prehospital PRIEST is related to the severity of illness and the definition of clinical outcome. Overall, a more severe condition corresponds to a higher optimal threshold for predicting death. The use of a fixed threshold value from previous studies may indicate good sensitivity at the expense of specificity [33]. Therefore, the threshold value determined based on the study object and patient’s condition has greater practical value than the fixed threshold value. Second, prehospital PRIEST helps prepare for treatment and transport in the prehospital setting. An exceeded threshold value indicates that these patients may be in a state of severe illness, multiple comorbidities, and delayed treatment. This finding also implies that the prehospital care workload increases, necessitating the preparation of adequate dedicated medical staff, medical equipment, and treatment measures [6]. Third, this risk stratification model based on prehospital PRIEST helps clinicians to rapidly establish a close connection between the prehospital and in-hospital phases of patients. For those with high prehospital PRIEST scores and associated risk factors such as fatigue, comorbidities, septic shock, and decreased SpO_2_, arrangements should be made for the ICU or high-dependency unit to ensure considerable attention and medical resources [34]. Fourth, compared with the other physiological parameters scores in this study, the PRIEST score, which includes 10 important physiological parameters closely related to the prognosis of patients with COVID-19, is worthy of improvement in clinical practice due to its superior accuracy [8,10,19,20]. For the convenience of practical application, we created some table styles for commonly used prehospital physiological parameters scores of patients with COVID-19. Thus, medical staff need only to tick to obtain the scores of patients. Five physiological parameter scores were obtained for each patient within 30 s in this study. Given the context of viral mutations, population vaccination, and the ­application of antiviral drugs, the predictive efficacy of PRIEST scores demands dynamic attention and validation.

## Limitations

This study has several limitations. First, due to the limited number of ICU beds in our hospital, accommodating the needs of all critically ill patients during the epidemic was impossible, potentially leading to adverse effects on their prognosis. Second, for patients with consciousness disorders, the description of symptoms by the patients and their relatives may have deviations and errors due to the subjectivity of clinical symptoms. Third, the days from the onset of symptom to admission vary due to subjective reasons given by the patients. Although this ensures the authenticity of this study, it may have affected the homogeneity of the observation baseline. Fourth, this research was limited by its single-center and retrospective nature, and lacked external validation. We eagerly anticipate conducting multicenter and well-designed prospective studies in the future.

## Conclusions

As a modification of the NEWS2, the prehospital PRIEST can accurately, quickly, and conveniently predict the 30-day mortality of COVID-19 patients with pneumonia. The prehospital PRIEST score was strongly correlated with the basic condition and prehospital care workload of these patients. PRIEST, which encompassed 10 important physiological parameters and served as a beneficial supplement to the guidelines of NIH, was appropriate for guiding the initial risk stratification and treatment of these patients during the prehospital phase.

## Data Availability

The datasets used or analyzed during the current study are available from the first author or corresponding author upon reasonable request. If you need supporting data, you can contact us at any time. Email: gemini_lee525@126.com; suhaibin302@163.com.
